# 176. Remdesivir for Post-Exposure Prophylaxis of Marburg Virus Disease: A Cohort Study Assessing Safety, Clinical, and Immunologic Outcomes

**DOI:** 10.1093/ofid/ofaf695.006

**Published:** 2026-01-11

**Authors:** Tsion Firew, Anna Dobbins, Espoir Hakizimana, Appolinaire Manirafasha, Vanessa Nadine Ineza, Zerihun Abebe, Kara L Neil, John Baptist Nkuranga, Janvier Ndayambaje, Rafiki Gatera, Claude Mambo Muvunyi, Edson Rwagasore, Jean Claude S Ngabonziza, Menelas Nkeshimana, Yvan Butera, Sabin Nzanzimana, Ryan Westergaard

**Affiliations:** Africa, Health Sciences University, Kigali, Rwanda King Faisal Hospital Rwanda, Kigali, Rwanda and New York University, New York, USA, KG 288, Kigali, Rwanda; Rwanda Ministry of Health, Kigali, Kigali, Rwanda; Africa, Health Sciences University, Kigali, Rwanda King Faisal Hospital Rwanda, Kigali, Rwanda, Kigali, Kigali, Rwanda; King Faisal Hospital Rwanda, Kigali, Rwanda, Kigali, Kigali, Rwanda; King Faisal Hospital Rwanda, Kigali, Rwanda, Kigali, Kigali, Rwanda; King Faisal Hospital Rwanda, Kigali, Rwanda, Kigali, Kigali, Rwanda; Africa, Health Sciences University, Kigali, Rwanda and King Faisal Hospital Rwanda, Kigali, Rwanda, Kigali, Kigali, Rwanda; King Faisal Hospital Rwanda, Kigali, Rwanda, Kigali, Kigali, Rwanda; Africa, Health Sciences University, Kigali, Rwanda King Faisal Hospital Rwanda, Kigali, Rwanda, Kigali, Kigali, Rwanda; King Faisal Hospital Rwanda, Kigali, Rwanda, Kigali, Kigali, Rwanda; RWANDA BIOMEDICAL CENTER, KIGALI, Kigali, Rwanda; Rwanda Biomedical Center, Kigali, Rwanda, Kigali, Kigali, Rwanda; Rwanda Biomedical Center, Kigali, Rwanda, Kigali, Kigali, Rwanda; Ministry of Health, Kigali, Rwanda, Kigali, Kigali, Rwanda; Ministry of Health Rwanda, KIGALI, Kigali, Rwanda; Ministry of Health Rwanda, KIGALI, Kigali, Rwanda; University of Wisconsin School of Medicine and Public Health, Madison, WI

## Abstract

**Background:**

Marburg Virus Disease (MVD) outbreaks have mortality rates up to 90% and disproportionately affect healthcare workers (HCWs). Although no treatments are approved, remdesivir, an antiviral drug that inhibits RNA-dependent RNA polymerase, was shown to protect against MVD in animal models. This study aims to evaluate the safety, clinical, and laboratory outcomes among HCWs who received a 3-day course of remdesivir following suspected MVD exposure during the 2024 outbreak in Rwanda.

**Table 1:**
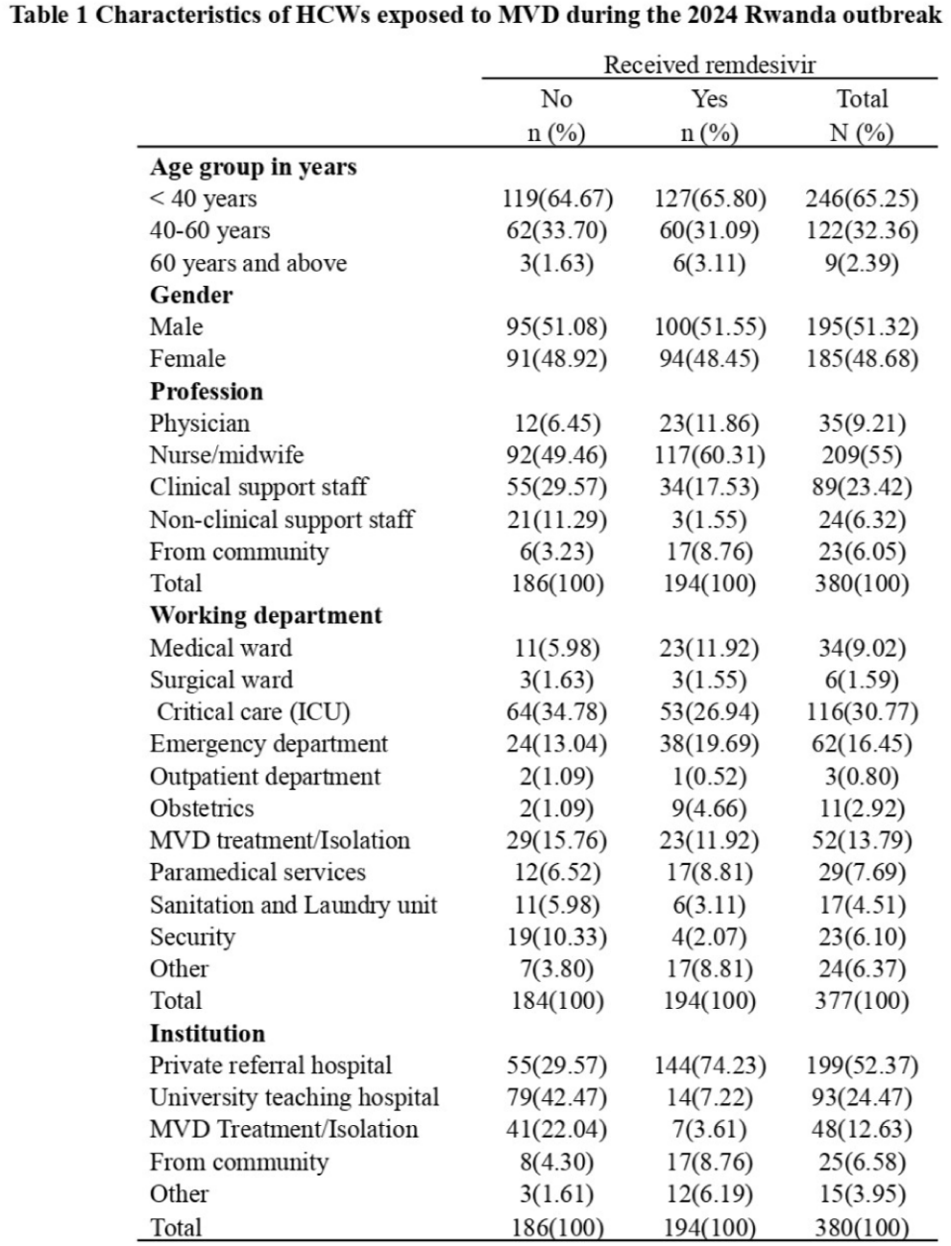
Characteristics of HCWs exposed to MVD during the 2024 Rwanda outbreak

**Table 2:**
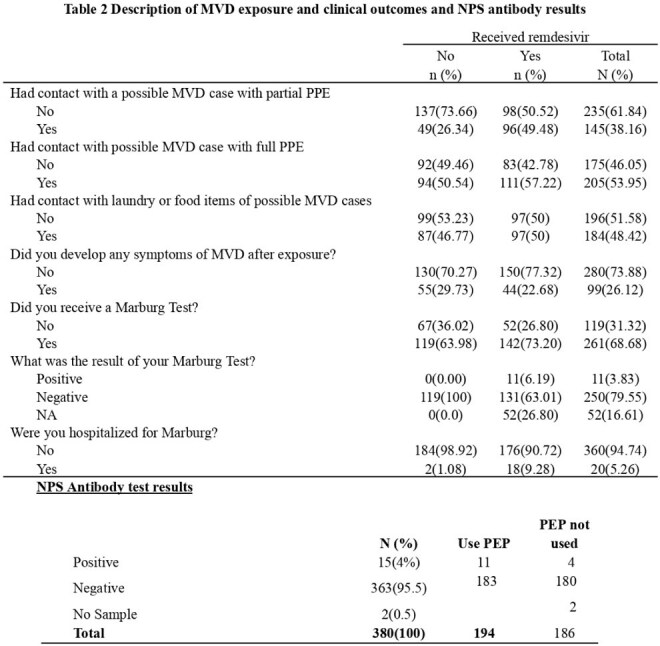
Description of MVD exposure and clinical outcomes and NPS antibody results

**Methods:**

Between September 27 and October 30, 2024, intravenous remdesivir (200 mg on Day 1, 100 mg on Days 2-3) was offered as post-exposure prophylaxis (PEP) to HCW who had worked at least one shift in MVD-affected units.Liver function tests (LFTs) and symptom assessments were performed pre- and post-remdesivir infusion. In March 2025, eligible HCWs were surveyed about post-treatment symptoms and provided blood samples for Marburg virus-specific antibody testing.CT values and incubation periods from MVD-positive individuals without PEP (Rwanda Biomedical Center) were compared to MVD-positive PEP participants in this study. Analyses used STATA v28.

**Table 3.**
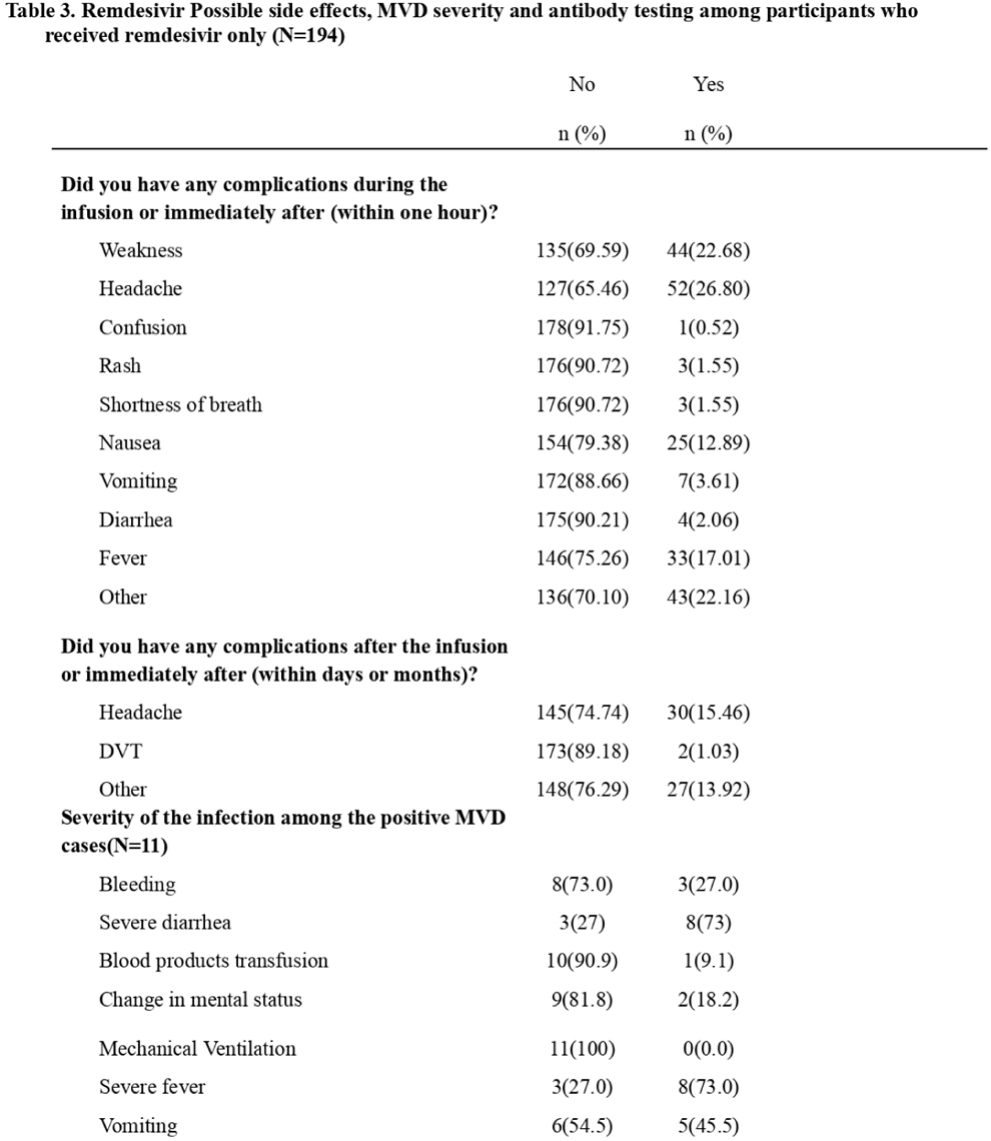
Remdesivir Possible side effects, MVD severity and antibody testing among participants who received remdesivir only (N=194)

**Graph 1.**
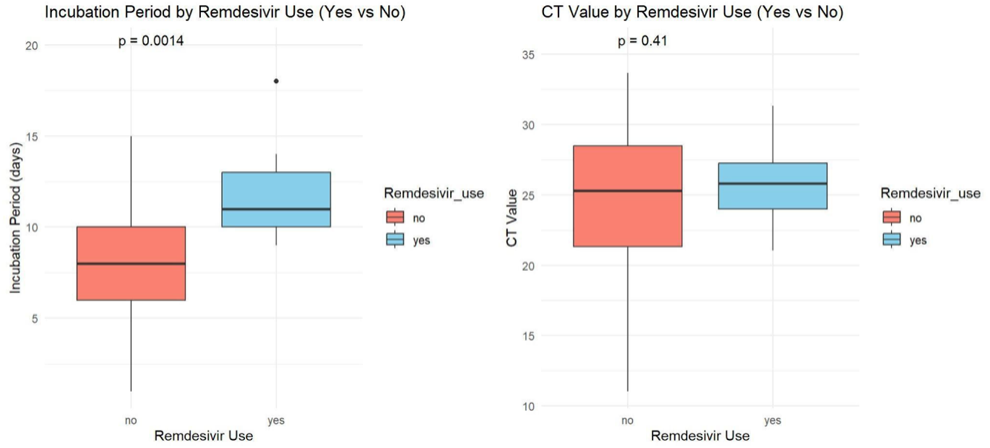
Box plot of CT values and incubation Period among Remdesivir use status

**Results:**

By July 14, 2025, 380 HCWs were enrolled: 194 (51%) received PEP and 186 (49%) declined. Participant characteristics are shown in Table 1. Overall, 261 (68.7%) reported MVD testing; 20 (5.3%) were hospitalized with MVD-like symptoms, and 11 (3.2%) tested positive. MVD exposure risks are presented in Table 2.15 HCWs (4%) developed MVD-specific antibodies. Table 3 summarizes self-reported complications and MVD severity. A significant difference in incubation period was observed between PEP recipients (median: 11 vs. 8 days; *P* = 0.0014).Seroprevalence was higher in the PEP group (5.6%) than in the non-PEP group (2.2%). CT values were similar (median: 26.29 vs. 25.38; *P* = 0.41), with less variation in the PEP group (Graph 1). LFTs showed no notable changes pre- and post-remdesivir.

**Conclusion:**

Remdesivir appears safe as post-exposure prophylaxis (PEP) for HCWs during MVD outbreaks.findings suggest that remdesivir may lead to a clinically and statistically significant extension of the incubation period, as well as a modest reduction in viral load at the time of diagnosis. However, controlled clinical trials are needed to confirm its preventive efficacy.

**Disclosures:**

All Authors: No reported disclosures

